# Extracellular CIRP induces CD4CD8αα intraepithelial lymphocyte cytotoxicity in sepsis

**DOI:** 10.1186/s10020-024-00790-2

**Published:** 2024-02-01

**Authors:** Yuichi Akama, Atsushi Murao, Monowar Aziz, Ping Wang

**Affiliations:** 1 Center for Immunology and Inflammation, The Feinstein Institutes for Medical Research, 350 Community Dr, 11030 Manhasset, NY USA; 2 Departments of Surgery and Molecular Medicine, Zucker School of Medicine at Hofstra/Northwell, Manhasset, NY USA

**Keywords:** CD4CD8αα IEL, Intestine, Sepsis, eCIRP, Granzyme B, Perforin

## Abstract

**Background:**

In sepsis, intestinal barrier dysfunction is often caused by the uncontrolled death of intestinal epithelial cells (IECs). CD4CD8αα intraepithelial lymphocytes (IELs), a subtype of CD4^+^ T cells residing within the intestinal epithelium, exert cytotoxicity by producing granzyme B (GrB) and perforin (Prf). Extracellular cold-inducible RNA-binding protein (eCIRP) is a recently identified alarmin which stimulates TLR4 on immune cells to induce proinflammatory responses. Here, we hypothesized that eCIRP enhances CD4CD8αα IEL cytotoxicity and induces IEC death in sepsis.

**Methods:**

We subjected wild-type (WT) and CIRP^−/−^ mice to sepsis by cecal ligation and puncture (CLP) and collected the small intestines to isolate IELs. The expression of GrB and Prf in CD4CD8αα IELs was assessed by flow cytometry. IELs isolated from WT and TLR4^−/−^ mice were challenged with recombinant mouse CIRP (eCIRP) and assessed the expression of GrB and Prf in CD4CD8αα by flow cytometry. Organoid-derived IECs were co-cultured with eCIRP-treated CD4CD8αα cells in the presence/absence of GrB and Prf inhibitors and assessed IEC death by flow cytometry.

**Results:**

We found a significant increase in the expression of GrB and Prf in CD4CD8αα IELs of septic mice compared to sham mice. We found that GrB and Prf levels in CD4CD8αα IELs were increased in the small intestines of WT septic mice, while CD4CD8αα IELs of CIRP^−/−^ mice did not show an increase in those cytotoxic granules after sepsis. We found that eCIRP upregulated GrB and Prf in CD4CD8αα IELs isolated from WT mice but not from TLR4^−/−^ mice. Furthermore, we also revealed that eCIRP-treated CD4CD8αα cells induced organoid-derived IEC death, which was mitigated by GrB and Prf inhibitors. Finally, histological analysis of septic mice revealed that CIRP^−/−^ mice were protected from tissue injury and cell death in the small intestines compared to WT mice.

**Conclusion:**

In sepsis, the cytotoxicity initiated by the eCIRP/TLR4 axis in CD4CD8αα IELs is associated with intestinal epithelial cell (IEC) death, which could lead to gut injury.

**Supplementary Information:**

The online version contains supplementary material available at 10.1186/s10020-024-00790-2.

## Introduction

Sepsis is a life-threatening organ dysfunction caused by a dysregulated host response to infection (Singer et al. 2016). It is known that intestinal epithelial cell (IEC) death can be induced in patients with sepsis, leading to intestinal barrier dysfunction (Mandal et al. 2018; Haussner et al. 2019). Recent studies have shown that experimental prevention of IEC death improved the survival rate of septic mice (Mittal and Coopersmith 2014). Thus, it is indicated that the IEC death could be a potential therapeutic target for septic patients. Nevertheless, the mechanism through which sepsis induces cell death in the intestinal epithelium remains elusive. CD4CD8αα intraepithelial lymphocytes (IELs) are a subtype of CD4^+^ T cells that reside within the epithelium of the small and large intestines (Mucida et al. 2013). CD4CD8αα IELs are considered to be originated from CD4 and CD8 double-positive thymocytes and are functionally mature T cells, unlike the immature CD4^+^CD8^+^ T cells in the thymus (Mucida et al. 2013; Eberl and Littman 2004). A recent study has shown that commensal bacteria facilitated the differentiation of CD4^+^ T cells into CD4CD8αα IELs, which could explain why CD4CD8αα IELs are predominantly found in the small and large intestines (Bousbaine et al. 2022). CD4CD8αα IELs exert cytotoxicity by secreting cytolytic granules, granzyme B (GrB) and perforin (Prf) (Mucida et al. 2013). It is known that CD4CD8αα IELs have plasticity, and their differentiation is closely related to the surrounding environment, such as intestinal microbiota (Bousbaine et al. 2022; Sujino et al. 2016; Cervantes-Barragan et al. 2017). In addition, the functional alteration of CD4CD8αα IELs has been observed under disease conditions. For instance, the biopsies of pediatric celiac disease patients showed an increased percentage of GrB^+^ CD4CD8αα IELs, suggesting their involvement in the pathogenesis of this disease (Costes et al. 2019). However, to our knowledge, the role of CD4CD8αα IELs in sepsis has not been studied.

Analysis of the pathological mechanism of extracellular cold-inducible RNA-binding protein (eCIRP) has been highlighted for its potential to elucidate the pathogenesis of sepsis (Aziz et al. 2019). Intracellular CIRP is an 18-kDa RNA chaperone regulating mRNA translation (Nishiyama et al. 1997). On the other hand, eCIRP serves as a damage-associated molecular pattern (DAMP) contributing to the pathogenesis of sepsis (Aziz et al. 2019; Qiang et al. 2013). eCIRP activates TLR4 on immune cells, such as macrophages, neutrophils, and T cells, to promote their proinflammatory responses (Aziz et al. 2019). eCIRP has also been shown to account for the dysfunction of multiple organs, including, the lungs, kidneys, liver, and intestines during sepsis (Aziz et al. 2019). In septic patients, eCIRP was elevated in circulation, and this elevation was correlated with disease severity. eCIRP affects the status of cells in different ways, including macrophage polarization, neutrophil heterogeneity, NETosis, T cell activation, and endothelial cell endoplasmic reticulum (ER) stress (Aziz et al. 2019; Zhou et al. 2020; Jin et al. 2023). Here, we hypothesized that eCIRP enhances CD4CD8αα IEL cytotoxicity and causes IEC death in sepsis. We found that CD4CD8αα IELs exhibited enhanced cytotoxicity in sepsis in an eCIRP-dependent manner. We have also demonstrated that eCIRP increased CD4CD8αα IEL cytotoxicity via TLR4 in vitro. Finally, cytotoxic CD4CD8αα IELs induced IEC death, which was associated with gut injury in sepsis.

## Materials and methods

### Mice

Specific pathogen-free male 8–12-week-old wild-type (WT) C57BL/6 mice were purchased from Charles River (Charles River, Wilmington, MA). C57BL/6 CIRP^−/−^ mice originally obtained from Dr. Jun Fujita (Kyoto University, Kyoto, Japan) and C57BL/6 TLR4^−/−^ mice obtained from Dr. Kevin Tracey (The Feinstein Institutes for Medical Research, Manhasset, NY) were bred and maintained in our facility. Mice were housed in a temperature-controlled room on 12 h light/dark cycles and provided standard laboratory chow and water.

### Murine model of polymicrobial sepsis

Mice were anesthetized with isoflurane (2%) in oxygen, and sepsis was induced by CLP(Qiang et al. 2013; Rittirsch et al. 2009). A 1.5 cm midline abdominal incision was made, and the cecum was exposed and ligated 1 cm proximal to the tip with a 4 − 0 silk suture. The cecum was perforated using a single through-and-through puncture using a 22-gauge needle midway between the ligation site and the tip of the cecum. A small amount of feces was gently squeezed out of the perforation site to ensure the patency of the punctures. The cecum was returned to the abdomen, and the abdominal wound was closed in two layers. Sham mice underwent abdominal incisions without ligation and puncture of the cecum.

### Isolation of IELs

IELs from small intestines were isolated with some modifications to the protocol described previously (Mucida et al. 2013; Moon et al. 2021). Briefly, small intestines were isolated and opened immediately after the removal of fat and Peyer’s patches. The contents were gently washed out with cold PBS. Sections of the small intestines (ileum) were incubated with 5 mM EDTA (Thermo Fisher Scientific, MA, USA) and 1 mM DTT (Thermo Fisher Scientific) in RPMI 1640 medium with 2% fetal bovine serum (FBS) at room temperature for 20 min to isolate the epithelial layer. Tissue pieces were then removed by a 100 μm mesh filter. Density gradient centrifugation was performed for the purification of IELs. 75% Percoll (Cytiva, MA, USA) was layered onto the cells suspended with 40% Percoll and centrifuged at 800 g for 20 min at 20℃. Cells at the interphase, which contained IELs, were collected and washed for the subsequent process.

### Flow cytometry

Single-cell suspensions were incubated with a combination of fluorescent conjugated antibodies (Abs) as follows: CD8α-BB515 (53 − 6.7, catalogue no.: 564,422, BD Biosciences, San Diego, California), CD8β-PE/Cy7 (YTS156.7.7, catalogue no.: 126,615, BioLegend, San Diego, CA), CD45-PerCP/Cy5.5 (I3/2.3, catalogue no.: 147,706, BioLegend), TCRβ-APC/Fire 750 (H57-597, catalogue no.: 109,245, BioLegend), TCRγδ-BV605 (GL3, catalogue no.: 118,129, BioLegend), CD4-BUV395 (RM4-5, catalogue no.: 568,375, BD Biosciences), Perforin-PE (S16009A, catalogue no.: 154,305, BioLegend), Foxp3-PE (MF23, catalogue no.: 560,414, Thermo Fisher Scientific), EpCAM-PE (G8.8, catalogue no.: 118,206, BioLegend), CD4-APC (GK1.5, catalogue no.: 100,412, BioLegend), and Granzyme B-APC (QA16A02, catalogue no.: 372,203, BioLegend). Cell viability was determined using a Zombie Aqua Fixable Viability Kit (BioLegend). Fc block (BioLegend) was used to prevent nonspecific antibody binding. The absolute number of cells was calculated by using Precision Count Beads (BioLegend). For the assessment of intracellular protein expression, cells were incubated with Brefeldin A (BioLegend), 50 ng/ml phorbol 12-myristate 13-acetate (PMA, Thermo Fisher Scientific), and 500 ng/ml ionomycin (Thermo Fisher Scientific) for 3 h at 37℃, followed by the staining with antibodies using Intracellular Fixation and Permeabilization Buffer Set (Thermo Fisher Scientific, Waltham, MA). Cells were analyzed on a FACSymphony flow cytometer (BD Biosciences), and data were analyzed using FlowJo software. CD4CD8αα IELs were identified as live CD45^+^TCRβ^+^TCRγδ^–^CD4^+^CD8α^+^CD8β^–^ population. Among the CD4^+^ IELs, the presence of Foxp3-expressing cells has been reported (Prakhar et al. 2021). However, we confirmed that CD4CD8αα IELs barely expressed Foxp3 (Figure S1).

### Analyzing flow cytometric data using the clustering method

The t-distributed stochastic neighbor embedding (t-SNE) algorithm was performed for unsupervised analysis of the entire flow cytometry dataset (12 samples) generated from sham and sepsis mice. The t-SNE algorithm was run on the DownSample of live CD45^+^ TCRβ^+^ CD4^+^cell populations [2500 cells, randomly selected from sham (*n* = 6) and sepsis (*n* = 6), 30,000 cells total)]. For the CD4 T cell subpopulation analysis, cells were gated on the live CD45^+^ TCRβ^+^ CD4^+^cells as follows: CD4CD8αα (CD8α^+^CD8β^−^), CD4CD8αβ (CD8α^+^CD8β^+^), CD4CD8^−^ (CD8α^−^CD8β^−^).

### Histological analysis of gut injury

Samples of the small intestines were collected at a site 3–5 cm proximal to the ileocecal valve 4 h after CLP. Pathological samples were fixed in 10% formalin and cut into 5-μm-thick sections, which were stained with hematoxylin and eosin (H&E) or a terminal deoxynucleotidyl transferase dUTP nick end labeling (TUNEL) assay kit (Roche Diagnostics, IN, USA). Villous heights and the TUNEL-positive cells were assessed by Eclipse Ti-S (Nikon, Tokyo, Japan) and LSM900 (Zeiss, Oberkochen, Germany) microscopes, respectively per high-power field (×200) randomly selected.

### CD4 T cell isolation and differentiation

Spleens were disrupted and homogenized by crushing and filtering through a sterile 70 μm nylon filter in complete RPMI 1640 medium, supplemented with 10% FBS, 1% penicillin-streptomycin, 10 mm HEPES, 2 mM L-glutamine, 1 mM sodium pyruvate, and 50 μM β-mercaptoethanol. Erythrocytes were eliminated using Lysing Buffer (BD Biosciences). Naïve CD4^+^ T cells were purified from splenic lymphocytes by immunomagnetic negative selection using EasySep Naïve CD4^+^ T cell Isolation Kits (STEMCELL Technologies, BC, Canada). In an incubator maintained at 37℃ with a humidified atmosphere of 5% CO_2_, naïve CD4^+^ T cells were differentiated into CD4CD8αα cells following the protocol described previously, (Wang and Ai 2021; Harada et al. 2022) with some modifications. Briefly, 1.5 × 10^5^ naïve splenic CD4^+^ T cells were cultured for 3 days in 96-flat plates pre-coated with 0.5 μg/ml of anti-CD3ε Ab (145-2C11, catalogue no.: 100,340, BioLegend) in complete RPMI 1640 with 0.5 μg/ml of anti-CD28 Ab (37.51, catalogue no.: 102,116, BioLegend), 1 nM PMA, 250 nM ionomycin, 0.5 μg/ml concanavalin A (ConA, Thermo Fisher Scientific), 10 nM retinoic acid (Thermo Fisher Scientific), 2 ng/ml TGFβ (BioLegend), and 20 ng/ml IFNγ (R&D systems). CD3/CD28 antibodies, PMA/Ionomycin, and Concanavalin A were used to maintain the cell viability and facilitate the differentiation and proliferation of the cells as previously described (Wang and Ai 2021; Wang et al. 2019). CD3/CD28 antibodies activate the TCR signaling pathway along with the co-stimulatory signals, and PMA/Ionomycin and Concanavalin A are known to amplify those cellular responses (Li and Kurlander 2010; Crawford et al. 2014; Ren et al. 2012; Oh-hora 2009; Boilard and Surette 2001). For Th1 differentiation, 10 ng/ml recombinant murine (rm) IL-12p70 (BioLegend) was added instead of retinoic acid, TGFβ, and IFNγ.

### eCIRP treatment of IELs

The purification of rmCIRP (denoted as eCIRP) has been described previously by our laboratory (Qiang et al. 2013). IELs were cultured for 3 h in 96-well flat bottom plates precoated with 5 μg/ml of anti-CD3ε Ab in complete RPMI 1640 with 5 μg/ml soluble anti-CD28 Ab in the presence of PBS or 5 μg/ml eCIRP.

### RNA isolation and real-time quantitative PCR

Total RNA was extracted from CD4CD8αα IELs, which were sorted by using BD FACSAria IIu (BD Biosciences), with RNeasy Micro Kit (QIAGEN, Hilden, Germany) according to the manufacturer’s instructions. Reverse transcription was performed using an iScript cDNA Synthesis Kit (Bio-Rad, CA, USA). Quantitative real-time PCR (qPCR) was performed using SYBR Green PCR Master Mix (Thermo Fisher Scientific) with a StepOnePlus Real-Time PCR system (Thermo Fisher Scientific) for measuring granzyme B and perforin mRNA expression. Mouse β-actin served as an endogenous control to normalize mRNA levels using the comparative Ct method. Primer sequences were as follows: β-Actin, forward 5´-CGTGAAAAGATGACCCAGATCA-3´ and reverse 5´- TGGTACGACCAGAGGCATACAG-3´; GrB, forward 5´-ACCCAAAGACCAAACGTGCT-3´ and reverse 5´-AGCAGGATCCATGTTGCTTC-3´; Prf, forward 5´-ACACAGTAGAGTGTCGCATGTAC-3´and reverse 5´-GTGGAGCTGTTAAAGTTGCGGG-3´.

### Organoid culture

The protocol used for the isolation of intestinal crypts and 3D organoid culture has been established and maintained as described previously (Sato and Clevers 2013). Briefly, primary small crypts were isolated from 8-12-week-old C57BL/6 mice. The small intestines were cut into 2–4 mm pieces and incubated with ice-cold 2.5 mM EDTA in PBS with 0.1% bovine serum albumin (BSA) for 20 min. The supernatant was discarded and ice-cold PBS with 0.1% BSA was added. The samples were pipetted up and down to isolate the crypts following a published protocol (Sato and Clevers 2013). The supernatant containing crypts was passed through a 70 μm cell strainer to remove debris and villi. Growth factor-reduced Matrigel (Corning, NY, USA) for the 3D unit basement was used with murine IntestiCult Organoid Growth Medium (STEMCELL Technologies) according to the manufacturer’s instructions. Crypts were resuspended in an appropriate volume of the pre-mixed Matrigel and IntestiCult, yielding approximately 10,000 to 15,000 crypts/ml. 70 μl of the crypts-Matrigel-IntestiCult mixture were carefully pipetted into the center of each well of a 24-well plate and incubated for 15 min at 37 °C for solidification. IntestiCult was added to cover the Matrigel for nourishment support and used for refreshment every 2 or 3 days. The initial medium was supplemented with Y-27,632 (10 μM). The organoids were cultured in a 37 °C, 5% CO_2_ incubator. Intestinal organoids were used 5 days after initiation of the culture process. All experiments were performed before the first passage.

### Assessment of CD4CD8αα cell cytotoxicity on intestinal epithelial cells

The intestinal organoids were collected and enzymatically dissociated into single cells by incubation with TrypLE Express Enzyme (Thermo Fisher Scientific) for 8 min at 37 °C. CD4CD8αα cells differentiated from naïve splenic CD4^+^ T cells were preincubated with 5 μg/ ml eCIRP or PBS for 4 h at 37℃ and were treated with GrB inhibitor (50 μM, Ac-IEPD-CHO, Sigma-Aldrich) and Prf inhibitor (100 nM, concanamycin A, MedChemExpress) or solvent control for the last 30 min. After washing CD4CD8αα cells, organoid-derived IECs and CD4CD8αα cells were then cocultured in the presence of 5 μg/ml anti-CD3ε Ab for 4 h (IECs:CD4CD8αα cells = 1:40). The cell death of CD45^−^EpCAM^+^ epithelial cells was evaluated by flow cytometry. When determining the ratio of lymphocytes to IECs in our study, we referred to previous publications, where a similar E (effector):T (target) ratio was used (Gillissen et al. 2016; Liu et al. 2007; Wu et al. 2018). One publication also suggests optimizing E/T ratio between 1:6.25–100 (Broussas et al. 2013).

### Statistical analysis

Statistical analysis was performed using Prism 10 (GraphPad Software, San Diego, CA), and *p* < 0.05 was considered statistically significant. The values for the mean and the standard error of the mean are presented. ANOVA was used for one-way comparison among multiple groups, and the significance was determined by the Tukey method. The Student t-test was applied for two-group comparisons. All experiments were repeated at least two times.

## Results

### CD4CD8αα IELs exhibit enhanced cytotoxicity in septic mice

**Fig. 1:**
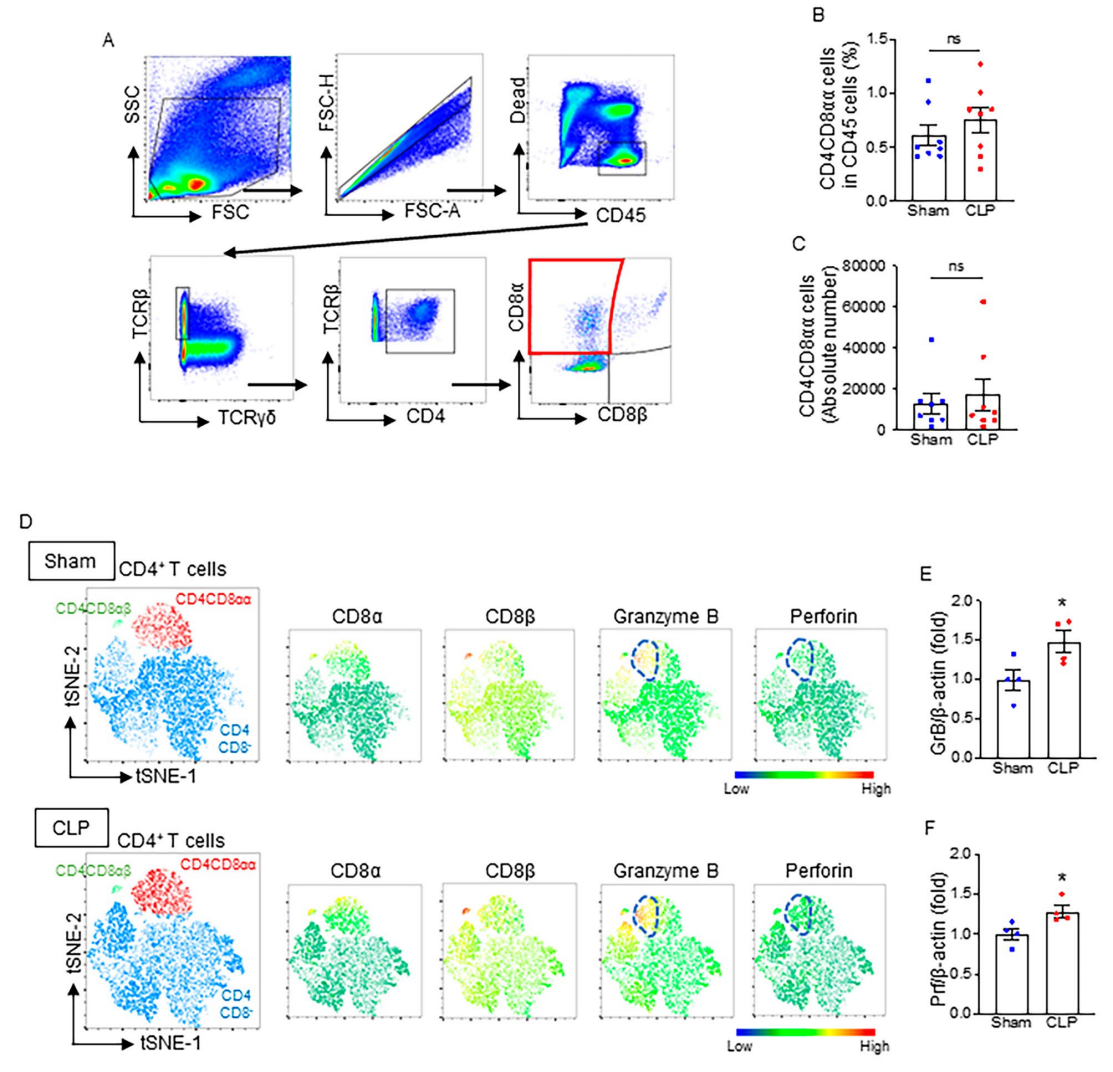
CD4CD8αα IELs predominantly exhibit enhanced cytotoxicity in septic mice. IELs were isolated from small intestines harvested from WT mice 4 h after sham or CLP surgery. (**A**) Representative gating strategy of flow cytometry plots for detecting live CD4CD8αα cells in IELs. (**B**) Percentage and (**C**) absolute number of CD4CD8αα IELs isolated from sham and CLP mice. Experiments were performed 3 times, and all data were used for analysis. Data represent the mean ± SEM (*n* = 5/group). The groups were compared by Student’s t-test. (**D**) Unsupervised clusterization of live TCRβ^+^CD4^+^ cells from the flow cytometry dataset of IELs in sham and sepsis mice was conducted using the t-distributed stochastic neighbor embedding (t-SNE) algorithm (each group is *n* = 15,000 from 6 mice). Heatmap density plots of CD8α, CD8β, GrB, and Prf were generated according to the median fluorescence intensity (MFI). Areas surrounded by dashed lines in CD4CD8αα population denote where GrB and Prf are highly expressed, and their intensities and cell numbers are increased after sepsis. CD4CD8αα IELs were isolated by FACS to determine the mRNA levels of (**E**) GrB and (**F**) Prf. Experiments were performed 2 times, and all data were used for analysis. Data represent the mean ± SEM (*n* = 4/group). The groups were compared by Student’s t-test. *p < 0.05 vs. sham

We induced sepsis in WT mice and harvested the small intestines 4 h after CLP to isolate IELs. First, we evaluated the changes in the frequency and number of CD4CD8αα IELs by flow cytometry. We found no significant change in the percentage or absolute count of CD4CD8αα IELs in CLP mice (Fig. 1A-C). We used the t-distributed stochastic neighbor embedding (t-SNE) algorithm to perform an unsupervised clusterization of the total CD4^+^ IEL population. Three CD4^+^ IEL subsets were identified, i.e., CD4CD8αα, CD4CD8αβ, and CD4CD8^−^ cells (Fig. 1D). We also evaluated the cytotoxicity of CD4^+^ IELs. Expression intensity revealed that GrB and Prf-expressing cells were mainly CD4CD8αα cells among the total CD4^+^ IELs (Fig. 1D). Moreover, the levels of GrB and Prf in CD4CD8αα IELs were increased in sepsis, as indicated by the highlighted area in the heatmap plots (Fig. 1D). We also found that mRNA levels of GrB and Prf were significantly increased in CD4CD8αα IELs after CLP (Fig. 1E, F). These data indicate that CD4CD8αα IELs predominantly exhibit enhanced cytotoxicity in septic mice.

### CIRP deficiency attenuates CD4CD8αα IEL cytotoxicity in septic mice

**Fig. 2:**
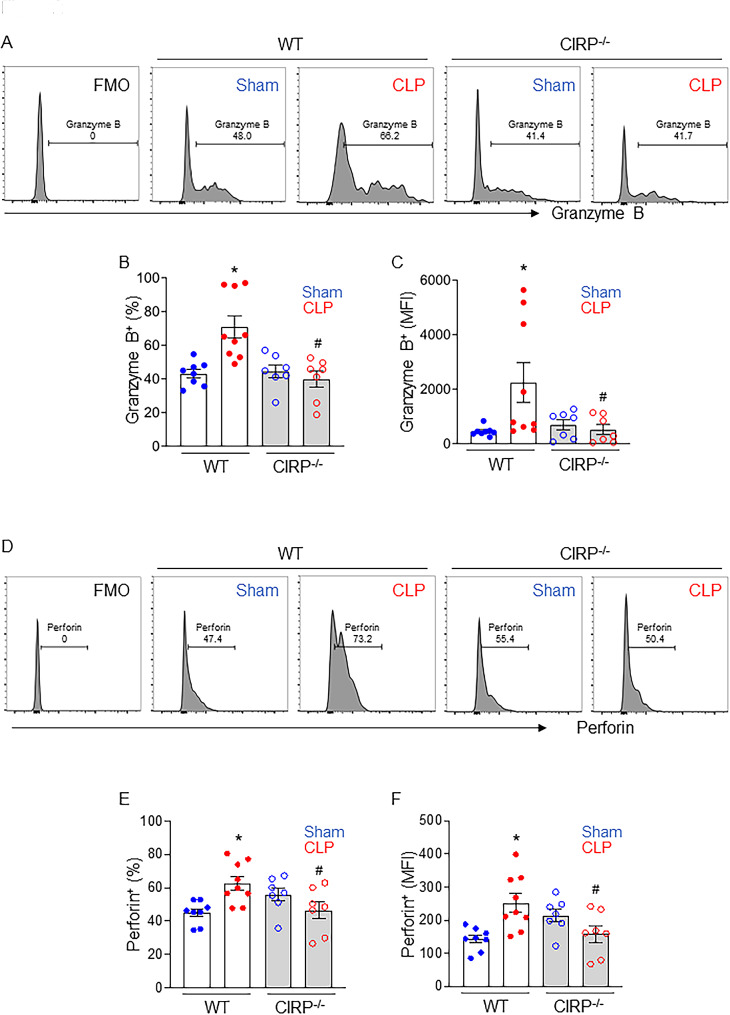
CIRP deficiency attenuates CD4CD8αα IEL cytotoxicity in septic mice. WT and CIRP−/− mice were subjected to sham or CLP and small intestine samples were collected 4 h after the surgery to assess cytotoxic granule levels of CD4CD8αα IELs using flow cytometry. (A, D) PercentageRepresentative histogram, (B, E) MFIpercentage and (C, F) histogramMFI of (A-C) GrB and (D-F) Prf expressions in CD4CD8αα IELs are shown. Experiments were performed 3 times, and all data were used for analysis. Data represent the mean ± SEM (n = 7–9/group). The groups were compared by one-way ANOVA followed by a Tukey’s multiple comparisons test t. *p < 0.05 vs. WT sham, #p < 0.05 vs. WT CLP.

Next, we investigated whether eCIRP is required for the increased GrB and Prf production by CD4CD8αα IELs in sepsis. We induced CLP in WT and CIRP^−/−^ mice and isolated the IELs. The levels of cytotoxic granules, GrB (Fig. 2A-C) and Prf (Fig. 2D-F), in CD4CD8αα IELs were significantly increased after sepsis in WT mice. Interestingly, the expressions of these cytotoxic granules in CD4CD8αα IELs were not affected by sepsis in CIRP^−/−^ mice (Fig. 2A-F). These data indicate that eCIRP plays a crucial role with regarding to the increase in GrB and Prf production of CD4CD8αα IELs during sepsis.

### eCIRP enhances cytotoxicity in CD4CD8αα IELs via TLR4

**Fig. 3:**
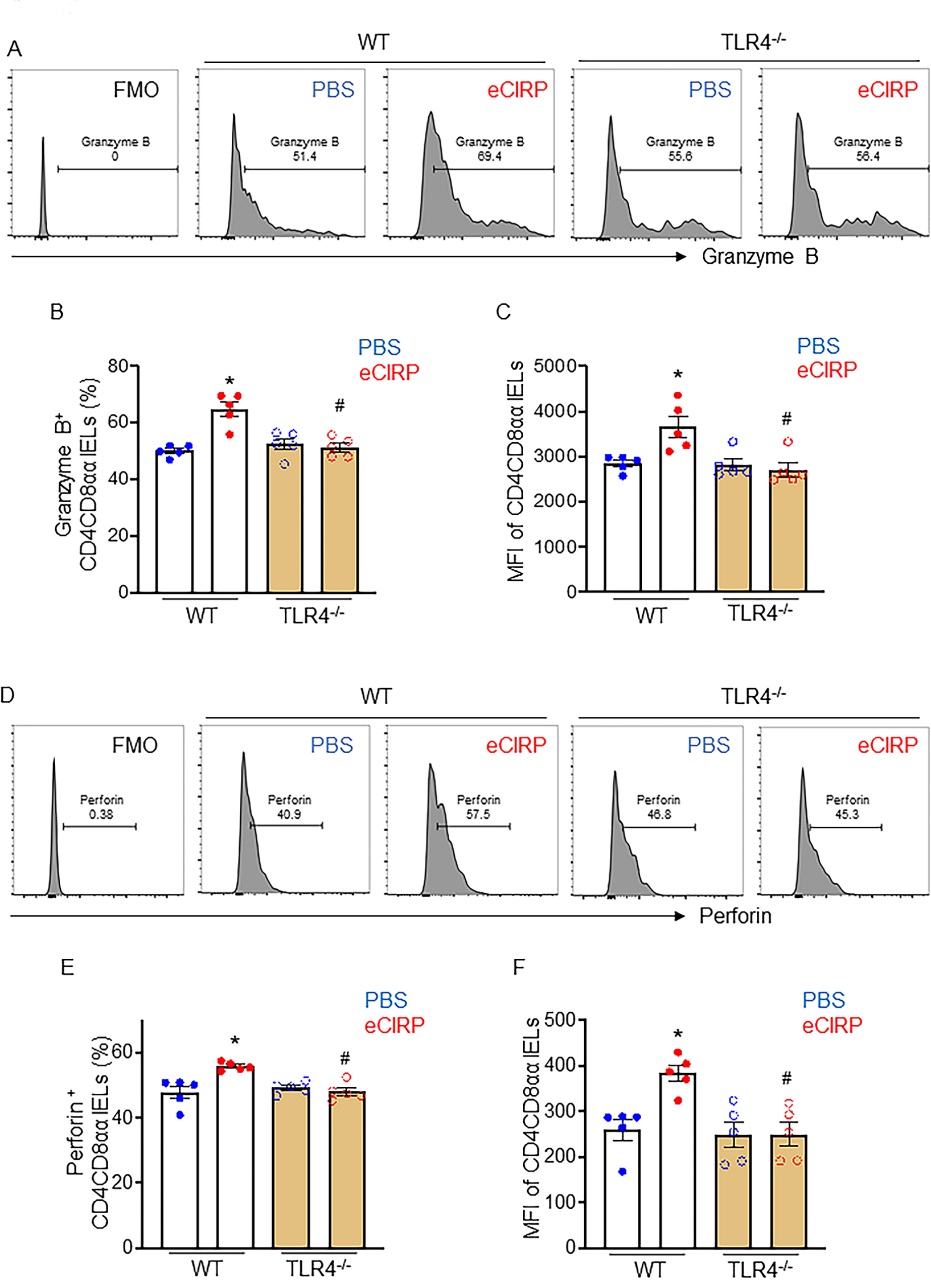
eCIRP enhances cytotoxicity in CD4CD8αα IELs via TLR4. IELs isolated from WT or TLR4^−/−^ mice were treated with PBS or eCIRP (5 μg/ml) for 3 h and cytotoxic granule levels of CD4CD8αα IELs were assessed by flow cytometry. (**A, D**) Representative histograms, (**B, E**) percentage and (**C, F**) MFI of (**A-C**) GrB and (**D-F**) Prf in CD4CD8αα IELs are shown. Experiments were performed 2 times, and all data were used for analysis. Data represent the mean ± SEM (*n* = 5/group). The groups were compared by one-way ANOVA followed by a Tukey’s multiple comparisons test. *p < 0.05 vs. WT PBS, ^#^*p* < 0.05 vs. WT eCIRP.

Subsequently, we challenged IELs with eCIRP in vitro. Treatment with eCIRP significantly increased the mRNA and protein levels of GrB (Fig. 3A-C, Figure S2A) and Prf (Fig. 3D-F, Figure S2B) in CD4CD8αα IELs derived from WT mice. We have previously reported that eCIRP activated CD4^+^ T cells in a TLR4-dependent manner (Bolognese et al. 2018), thus we assessed the involvement of TLR4 in eCIRP-induced CD4CD8αα IEL cytotoxicity by using IELs isolated from TLR4^−/−^ mice. On the contrary to WT CD4CD8αα IELs, eCIRP had no effect on the levels of GrB and Prf in CD4CD8αα IELs derived from TLR4^−/−^ mice (Fig. 3A-F), indicating that eCIRP-mediated cytotoxicity of CD4CD8αα IELs was induced via TLR4. Taken collectively, eCIRP enhances the production of GrB and Prf in CD4CD8αα IELs through TLR4.

### eCIRP-mediated cytotoxic CD4CD8αα cells induce intestinal epithelial cell death

**Fig. 4:**
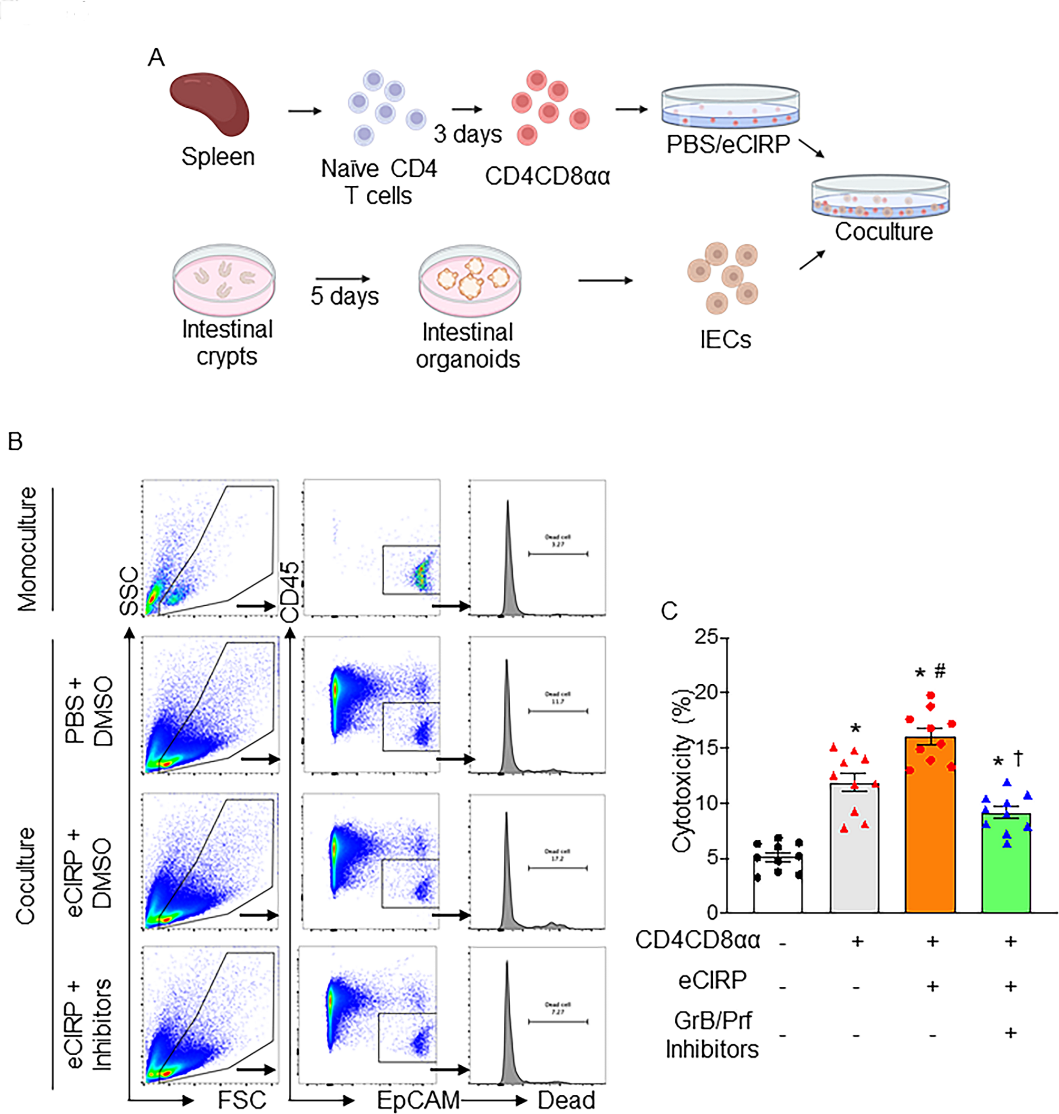
eCIRP-induced cytotoxic CD4CD8αα cells induce intestinal epithelial cell death. **(A)** A schematic of cytotoxicity assay using CD4CD8αα cells differentiated from splenic naïve CD4^+^ T cells and IECs derived from intestinal organoids. CD4CD8αα cells were pretreated with PBS or eCIRP for 4 h in the presence and absence of GrB (50 μM) and Prf (100 nM) inhibitors for the last 30 min. IECs and CD4CD8αα cells were then cocultured for 4 h, and cell death of IECs was evaluated by flow cytometry using a Zombie Aqua Fixable Viability Kit. (**B**) Representative flow cytometry plots and histogram showing CD45^−^EpCAM^+^ intestinal epithelial cells’ death rate. (**C**) Cytotoxicity of CD4CD8αα cells measured by the frequency of dead IECs. Experiments were performed 2 times, and all data were used for analysis. Data represent the mean ± SEM. The groups were compared by one-way ANOVA followed by a Tukey’s multiple comparisons test. *p < 0.05 vs. CD4CD8αα(-) eCIRP(-) inhibitors(-), ^#^*p* < 0.05 vs. CD4CD8αα(+) eCIRP(-) inhibitors(-), ^†^*p* < 0.05 vs. CD4CD8αα(+) eCIRP(+) inhibitors(-)

We then investigated the impact of cytotoxic CD4CD8αα IELs on IEC death. To evaluate the direct interaction between the two cell types, we developed an in vitro culture system. To obtain enough viable cells, CD4CD8αα cells were differentiated from splenic naïve CD4^+^ T cells using specific culture media (Fig. 4A). We validated that CD4^+^ T cells acquired CD8αα phenotype after incubation (Figure S3). IECs were isolated from intestinal organoids developed from intestinal crypts (Fig. 4A). CD4CD8αα cells, pretreated with either PBS or eCIRP in the presence or absence of GrB and Prf inhibitors, were then cocultured with IECs, and cell death of IECs was evaluated. eCIRP-treated CD4CD8αα cells induced significantly more IEC death than PBS-treated CD4CD8αα cells (Fig. 4B, C). However, GrB and Prf inhibitors significantly attenuated IEC death induced by eCIRP-treated CD4CD8αα cells (Fig. 4B, C). Utilizing organoid culture, we extended our validation of the in vitro co-culture results (Figure S4), confirming the role of eCIRP in facilitating the cytotoxicity of CD4CD8αα cells and their capacity to induce intestinal epithelial cell death in the context of sepsis. These data indicate that eCIRP-mediated cytotoxic CD4CD8αα cells directly induce IEC death, potentially contributing to intestinal injury.

### CIRP deficiency attenuates injury and cell death in intestinal epithelium of septic mice

**Fig. 5:**
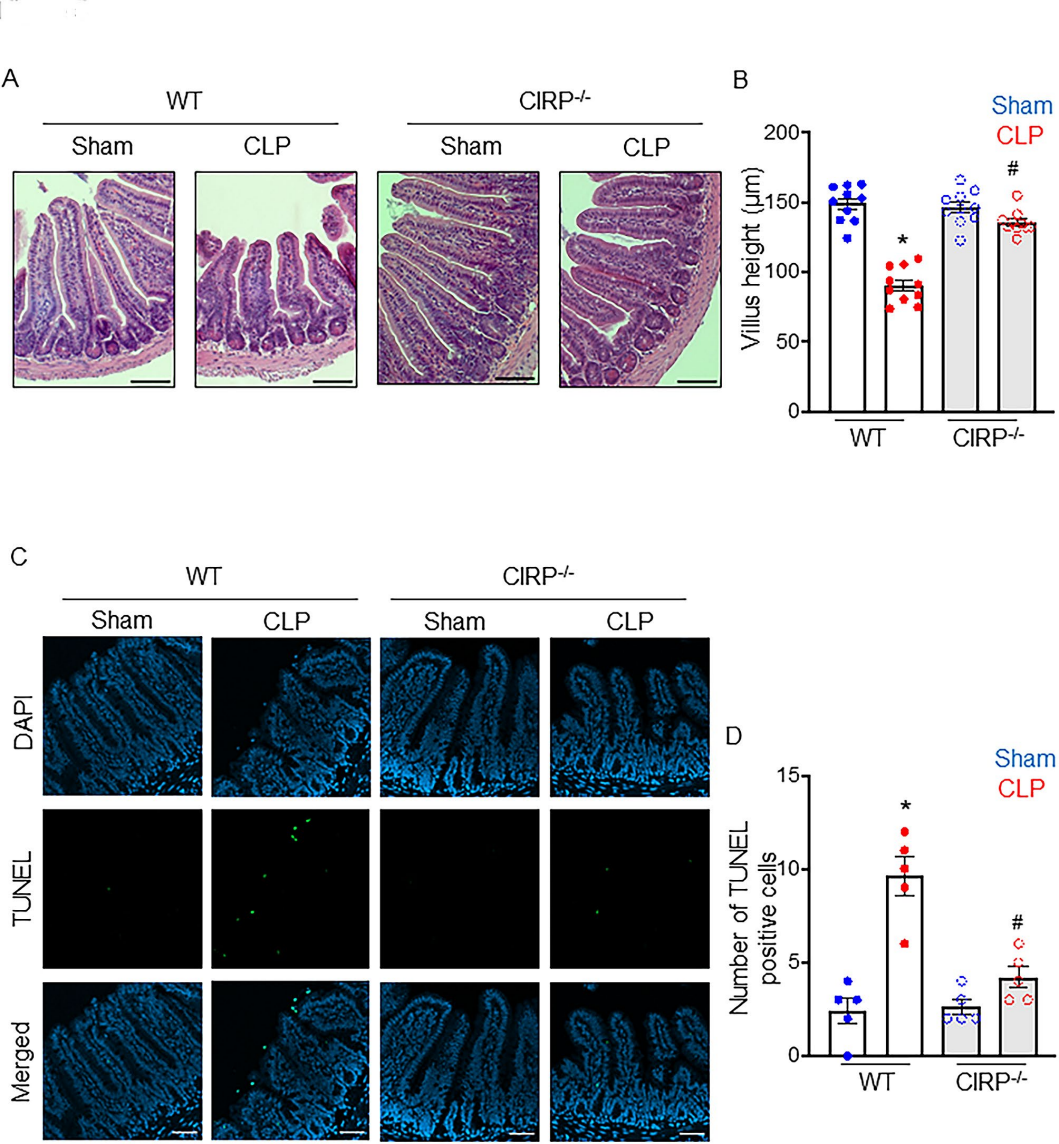
CIRP deficiency attenuates injury and cell death in intestinal epithelium of septic mice. Small intestines of WT or CIRP^−/−^ mice subjected to sham or CLP surgery were harvested for histological analysis. (**A**) Representative H&E images are shown at ×200 original magnification. Scale bars; 50 μm. (**B**) Villous height was measured. (**C**) Representative immunofluorescence images of TUNEL (green) staining and nuclear counterstaining (blue) are shown at ×200 original magnification. Scale bars; 50 μm. (**D**) Numbers of TUNEL^+^ cells in small intestinal tissues. Experiments were performed 2 times, and all data were used for analysis. A random selection of 3 high power fields (×200) were analyzed per mouse. Data represent the mean ± SEM (*n* = 5–10/ group). Scale bars; 50 μm. The groups were compared by one-way ANOVA followed by a Tukey’s multiple comparisons test. *p < 0.05 vs. WT sham, ^#^*p* < 0.05 vs. WT CLP.

**Fig. 6:**
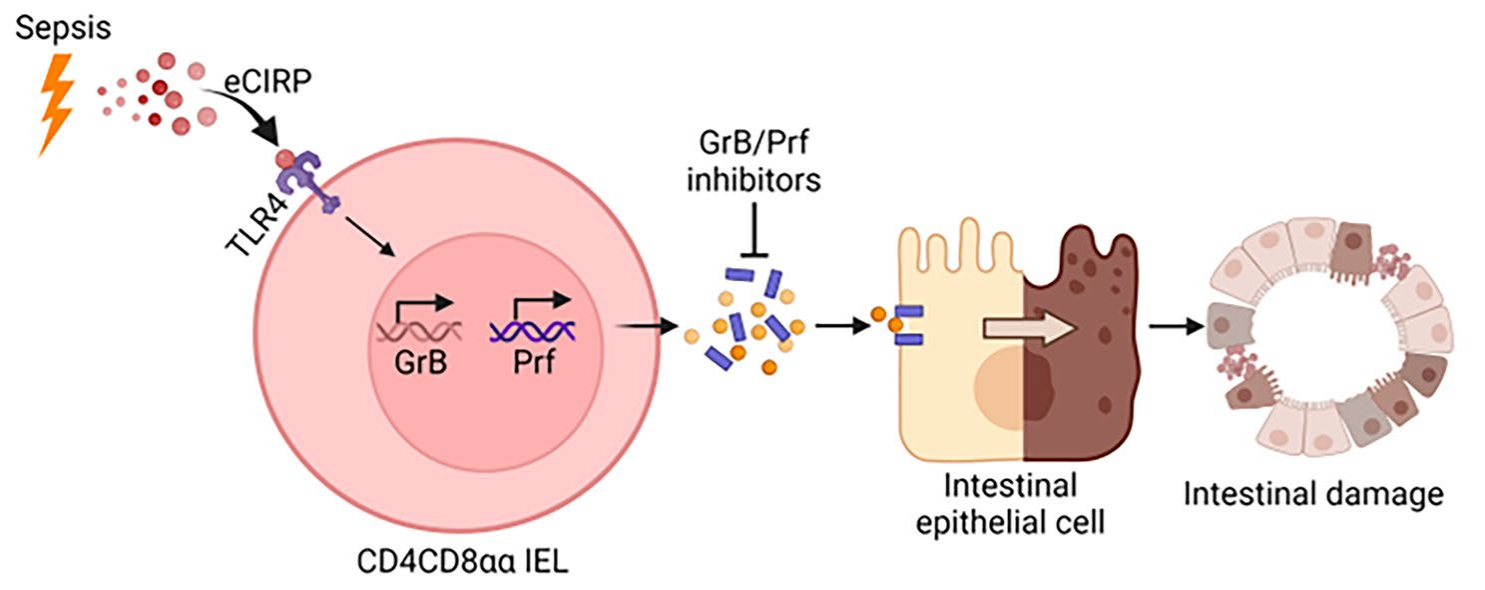
Summary of the findings eCIRP activates TLR4 to enhance the cytotoxicity of CD4CD8αα IELs as indicated by the increased production of GrB and Prf. Cytotoxic CD4CD8αα IELs induce IEC death, leading to intestinal injury

Finally, we performed pathological evaluation of the small intestines to assess the consequence of the preceding mechanisms initiated by eCIRP. We evaluated injury in intestinal epithelium by H&E staining in WT and CIRP^−/−^ mice subjected to sham or CLP surgery. H&E staining showed that the villi of WT mice were significantly shorter in CLP compared to sham, indicating that sepsis caused injury in intestinal epithelium of WT mice (Fig. 5A, B). However, CIRP^−/−^ mice were protected from epithelial damage as indicated by maintained villus height (Fig. 5A, B). Since we found that eCIRP-mediated cytotoxic CD4CD8αα cells induced IEC death, we subjected the intestinal tissues of WT and CIRP^−/−^ septic mice to TUNEL staining, which is known to reflect IEC death (Hu et al. 2019). The number of TUNEL-positive cells was significantly increased in WT septic mice, while CIRP^−/−^ mice did not show apparent changes in the number of TUNEL-positive cells after sepsis (Fig. 5C, D). These results indicate that eCIRP is implicated in a decrease in villous height and an increase in cell death of intestinal epithelium, which could reflect the increased cytotoxicity of CD4CD8αα IELs by eCIRP as shown earlier in this study (Fig. 6).

## Discussion

In the present study, we have demonstrated that eCIRP enhanced cytotoxicity through GrB and Prf production in CD4CD8αα IELs via TLR4 and caused intestinal epithelial cell death in sepsis. This finding would give insight into the pathogenesis of intestinal injury in sepsis, potentially leading to a novel therapeutic strategy for the disease. The precise role of CD4CD8αα IELs in gut immunity remains controversial and research in this area is ongoing (Zhou et al. 2019). It has been suggested that CD4CD8αα IELs can be cytotoxic or immunosuppressive depending on the conditions. CD4CD8αα IELs possess cytotoxicity as evidenced by the expression of effector molecules, such as GrB and Prf (Mucida et al. 2013; Zhou et al. 2019), which were the primary focus of our study in sepsis. Furthermore, we elucidated the novel role of eCIRP, a critical DAMP, in reshaping the phenotype of CD4CD8αα IELs, especially regarding their cytotoxicity.

Various intracellular contents are released during sepsis, acting as DAMPs that promote further inflammation and damage to organs (Denning et al. 2019). eCIRP is released in response to lipopolysaccharide (LPS) and hypoxia to serve as a DAMP to worsen the outcomes of sepsis. It has been shown that septic patients with elevated plasma levels of eCIRP had a poor prognosis (Qiang et al. 2013; Zhou et al. 2015; Gurien et al. 2020). These experimental and clinical findings indicate that eCIRP plays a significant role in the pathogenesis of sepsis. We have previously shown that eCIRP stimulated TLR4 on total CD4^+^ T cells to activate their functions (Qiang et al. 2013; Bolognese et al. 2018). Our recent study has also shown that eCIRP increases the mRNA expression of GrB in CD8^+^ T cells (Bolognese et al. 2018). Another study demonstrated that monocyte GrB can be upregulated in a TLR4/NF-κB-dependent manner. To our knowledge, there have not been studies elucidating the mechanism of cytotoxic granule upregulation in CD4CD8αα IELs. Here, we have demonstrated that the cytotoxicity of CD4CD8αα IELs can be enhanced in a similar mechanism as reported in other cell types, i.e., via the eCIRP-TLR4 axis.

It was classically thought that cell death could be divided into two forms, necrosis and apoptosis (Degterev and Yuan 2008). Necrosis is an uncontrolled cell death characterized by cell swelling and plasma membrane rupture, resulting in the massive release of proinflammatory molecules, such as DAMPs (Murao et al. 2021a, b). Apoptosis was traditionally regarded as a programmed cell death which does not cause the release of intracellular contents (Elmore 2007). However, it has recently been shown that apoptosis can also be immunogenic under certain conditions, such as irradiation or chemotherapy (He et al. 2018; Solari et al. 2020). Moreover, recent studies have discovered other types of programmed cell death, including, but not limited to, pyroptosis, necroptosis, and ferroptosis, all of which play important roles in sepsis. It is also known that GrB induces not only apoptosis but also other types of cell death, such as pyroptosis, in target cells. Here, we validated that cytotoxic CD4CD8αα IELs cause IEC death but did not specifically identify the type of cell death. In our septic mice, we evaluated cell death by TUNEL staining, which is commonly used to identify apoptotic cells but is also known to reflect other types of cell death. It would be of interest to assess the status of caspases and other cell death regulators in IECs affected by cytotoxic CD4CD8αα IELs to further delineate the underlying mechanisms of this interaction. While we did not use specific markers to identify TUNEL^+^ cells, recent studies have shown that gut TUNEL staining largely reflects IEC death (Dong et al. 2017; Gunther et al. 2015; Feng et al. 2006; Jilling et al. 2004). Furthermore, the localization of the TUNEL^+^ cells in our images matched with the area where IECs are the predominant cell population, specifically at the villous tip, from where these cells are expelled and cleared. The removal of dead IECs is another important aspect to be considered as the insufficient clearance of dead cells causes inflammation to further aggravate the intestinal barrier disruption, leading to the increased translocation of gut bacteria and septicemia. During sepsis, scavenging molecules that facilitate dead cell clearance, such as MFG-E8, have been shown to decrease in the body (Wu et al. 2022). Thus, the replenishment of scavenging molecules or synthetic drugs derived from those molecules have the potential to improve the outcomes of septic patients.

From the methodological aspect, CD4CD8αα IELs are a minor population, and IELs in general are susceptible to cell death ex vivo (James et al. 2020; Hoytema et al. 2017), thus it is difficult to perform culture experiments using primary CD4CD8αα IELs. While IL-15 extends IEL survival (Lai et al. 1999), it also provides CD4CD8αα IELs with the potential to produce IFN-γ, which is barely released at the steady state biologically (Mucida et al. 2013). To overcome these problems, we differentiated CD4CD8αα cells from naïve splenic CD4 cells to perform ex vivo experiments. This approach has been reported in multiple previous studies on CD4CD8αα IELs. We confirmed that these differentiated splenic CD4^+^ T cells indeed acquired CD8αα expression, which reflects the phenotype of CD4CD8αα IELs. The ex vivo culture of primary intestinal epithelial cells is also challenging. No definitive, reproducible, and robust primary small intestinal culture system has been developed. As an alternative approach, intestinal organoids have been utilized recently (Sato and Clevers 2013; Sato et al. 2009). Since organoids were formed inside the Matrigel of the 3D culture system, and CD4CD8αα cells could not reach inside the culture system, we dissociated organoids to make a single-cell suspension of IECs so that those two cell types could directly contact with each other. Taken together, in our culture system, we used differentiated splenic CD4CD8αα cells and intestinal organoid-derived cells to evaluate the direct causal impact of CD4CD8αα IELs on IECs.

While WT and CIRP-deficient mice were not littermates, these mice were housed in the same environment in our animal facility, minimizing the impact of any environmental factor to influence the results. Beyond the polymicrobial sepsis model induced by CLP, our prior studies have encompassed diverse models including LPS-induced endotoxemia, non-sterile inflammation models like gut ischemia/reperfusion, and eCIRP-induced acute lung injury (Zhou et al. 2023; Murao et al. 2021a, b; Khan et al. 2017; Cen et al. 2017). Across these systemic inflammation models, consistent findings revealed reduced inflammation and improved outcomes in CIRP^−/−^ mice, highlighting the detrimental role of eCIRP in exacerbating both sterile and non-sterile inflammation. Furthermore, in our present study, no significant differences in cytotoxic granule levels were observed in CD4CD8αα IELs between WT and CIRP^−/−^ mice under the normal condition. Recognizing the potential constraints arising from the breeding differences in our in vivo investigations, we also carried out in vitro experiments. In these experiments, we treated WT IELs with eCIRP to solidify the characterization of eCIRP as a pro-inflammatory molecule.

We acknowledge the limitations of our study. While we showed that eCIRP accounted for the increase in cytotoxicity of CD4CD8αα IELs and intestinal injury in septic mice in vivo and eCIRP-mediated cytotoxic CD4CD8αα cells caused IEC death in vitro, we did not directly validate the impact of cytotoxic CD4CD8αα IELs on IEC death or intestinal injury in septic mice. Even though it is extremely challenging with the current technologies, future studies using mice specifically deficient in CD4CD8αα IELs or conditional knockout of cytotoxic granules in CD4CD8αα IELs are necessary to rigorously validate the contribution of CD4CD8αα IELs to intestinal injury in sepsis. In addition, this study does not contain any human data. To date, there have been extremely limited reports on CD4CD8αα IELs in humans. One study has shown that GrB^+^ cell population was increased from 2 to 86% within CD4CD8𝛼𝛼 IELs in the biopsies of pediatric celiac disease patients, but the absolute number of those cells was not evaluated in that study. Even though it is difficult to obtain samples containing CD4CD8𝛼𝛼 IELs from septic patients due to the localization of this cell type to the intestinal epithelium, human studies are necessary to strictly determine their roles in the pathophysiology of this disease. Further, we did not utilize any therapeutic interventions in this study. We have recently developed synthetic inhibitors targeting eCIRP, which have shown beneficial effects on septic mice (Bolognese et al. 2018; Khan et al. 2017; Denning et al. 2020; Tan et al. 2020). It would be interesting to investigate whether the pharmacological inhibition of eCIRP can attenuate cytotoxicity in CD4CD8αα IELs and prevent IEC death to improve intestinal injury in sepsis.

In summary, we have found that eCIRP-mediated cytotoxic CD4CD8αα IELs induced IEC death, which was associated with damage of the intestinal epithelium in sepsis. Although further investigations of the underlying mechanisms and human studies are needed, our findings may contribute to filling the critical knowledge gap surrounding immune homeostasis of the intestine in sepsis that has hampered the development of new therapeutic strategies.

### Electronic supplementary material

Below is the link to the electronic supplementary material.

Supplementary Material 1

## Data Availability

All the data will be available from the corresponding author upon request.

## Abbreviations

DAMP: Damage-associated molecular pattern
eCIRP: Extracellular cold-inducible RNA-binding protein
GrB: Granzyme B
IEC: Intestinal epithelial cell
IEL: Intraepithelial lymphocyte
Prf: Perforin
