# Supplementary material for: Extracellular CIRP induces CD4CD8αα intraepithelial lymphocyte cytotoxicity in sepsis
Source: Mol Med. 2024 Feb 1;30:17. doi: 10.1186/s10020-024-00790-2 (PMC10835974; doi:10.1186/s10020-024-00790-2)
Supplement: Supplementary file 1 — Supplementary Material 1 [file 10020_2024_790_MOESM1_ESM.pdf]

Supplemental Fig 1

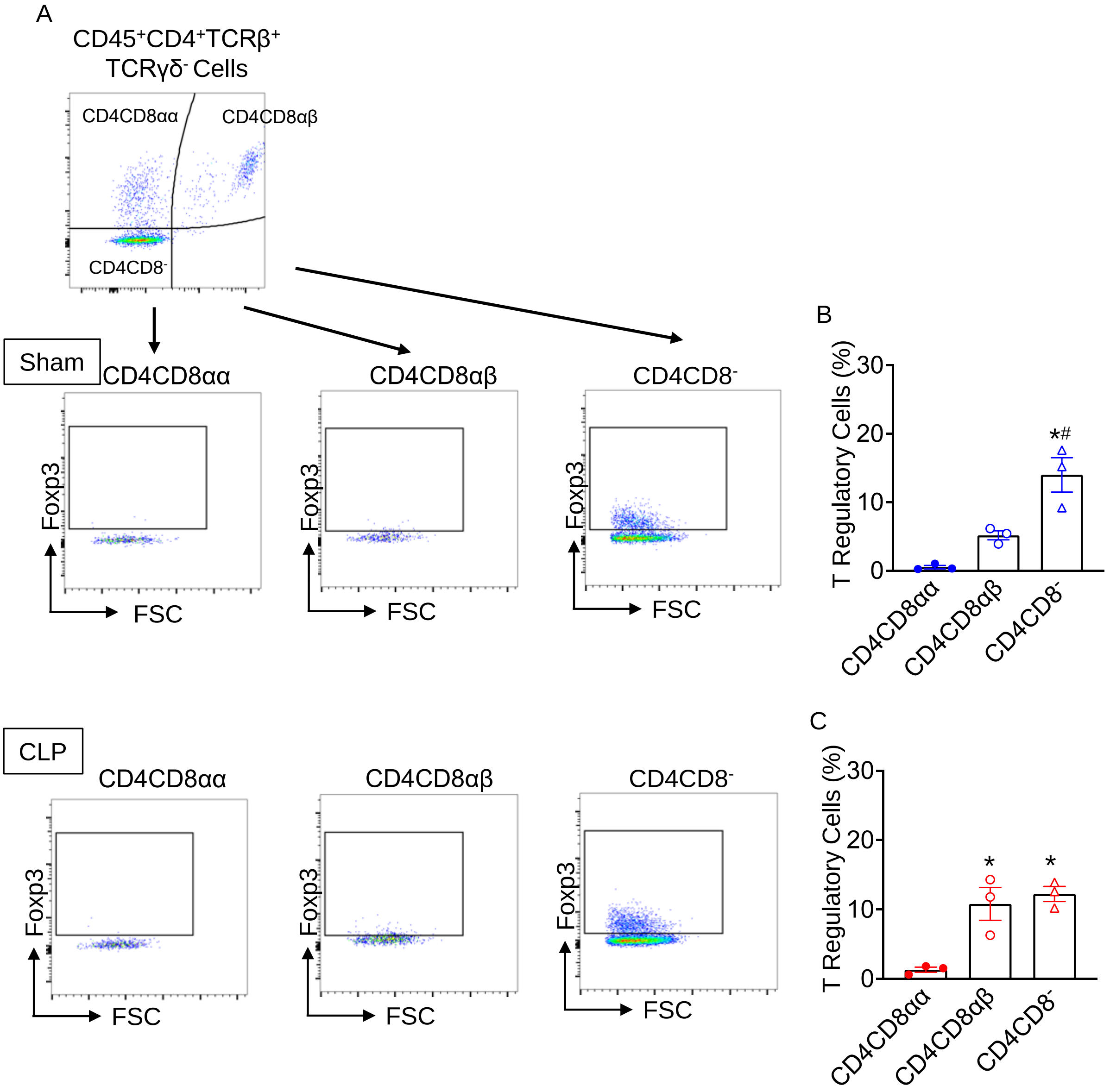

Supplemental Figure 1. CD4CD8αα IELs barely express Foxp3.

The proportion of Foxp3-expressing T regulatory cells in CD4 IEL subsets. Experiments were performed 2 times, and all data were used for analysis. Data represent the mean ± SEM (n = 3/ group). The groups were compared by one-way ANOVA followed by a Tukey's multiple comparisons. *$p < 0.05$ vs. CD4CD8αα, $^{\#}p < 0.05$ vs. CD4CD8αβ.

Supplemental Fig 2

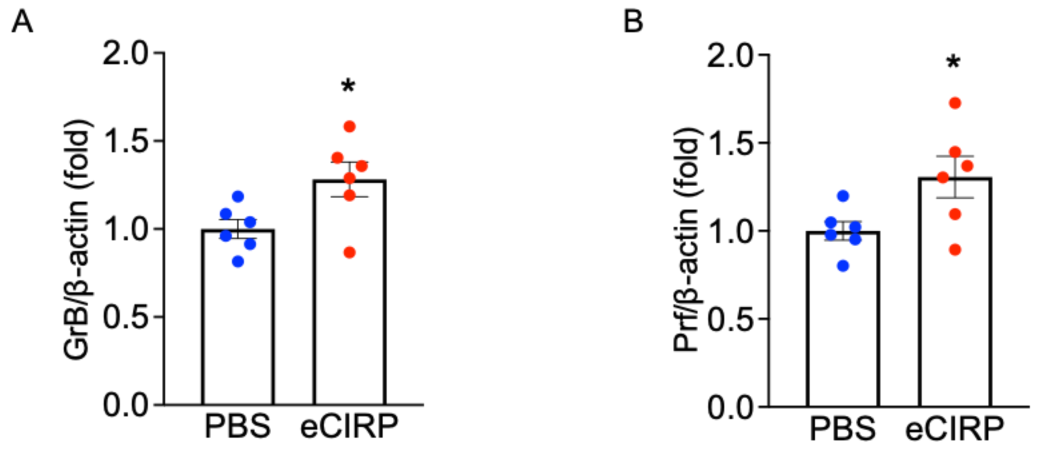

Supplemental Figure 2. GrB and Prf mRNA levels of eCIRP-treated CD4CD8αα IELs. IELs were treated with PBS or eCIRP (5 μg/ml) for 3 h. CD4CD8αα IELs were isolated by FACS and mRNA levels of **(A)** GrB and **(B)** Prf were assessed by qPCR. Experiments were performed 3 times, and all data were used for analysis. Data represent the mean ± SEM (n=6/group). The groups were compared by Student's t-test. *$p < 0.05$ vs. PBS.

Supplemental Fig 3

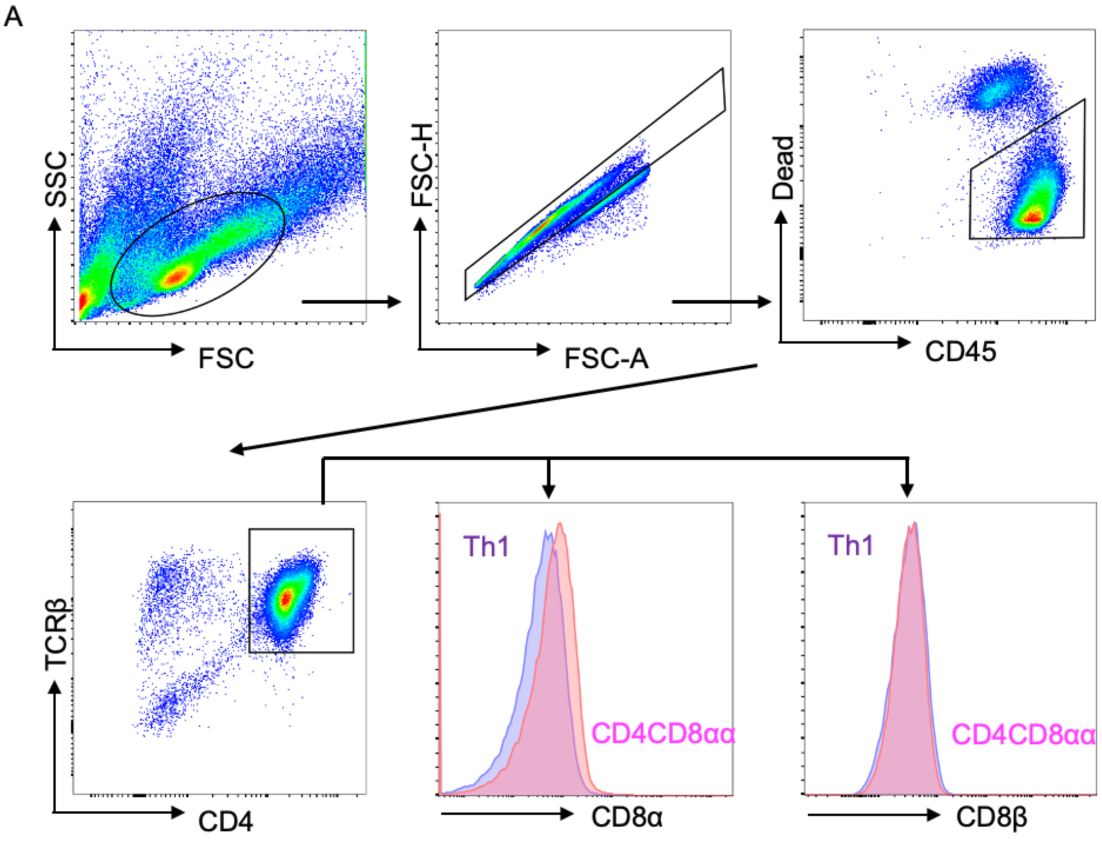

Supplemental Figure 3. Characteristics of differentiated CD4⁺ splenocytes.

Representative flow cytometry plots and histograms showing the expressions of CD8α and CD8β in live $CD45^{+}TCR\beta^{+}CD4^{+}CD8\beta^{-}$ cells under CD4CD8αα- and Th1-inducing conditions.

Supplemental Fig 4

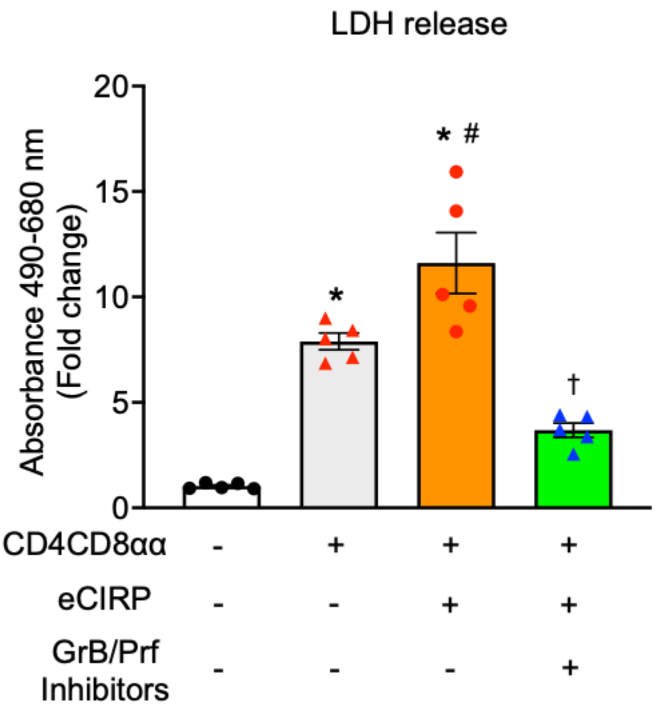

Supplemental Figure 4. Assessment of CD4CD8αα cell cytotoxicity by LDH levels. Cytotoxic assay of IEC/CD4CD8αα cell coculture treated with eCIRP and Grb/Prf inhibitors measured by LDH levels using CyQUANT LDH Cytotoxicity Assay (Thermo Fisher Scientific). *$p < 0.05$ vs. CD4CD8αα(-) eCIRP(-) inhibitors(-), $^{\#}p < 0.05$ vs. CD4CD8αα(+) eCIRP(-) inhibitors(-), †$p < 0.05$ vs. CD4CD8αα(+) eCIRP(+) inhibitors(-).
